# Playing Hard with Si: Challenges and Opportunities for New Materials in Radiation Therapy Dosimetry

**DOI:** 10.1002/adma.202508478

**Published:** 2025-07-24

**Authors:** James Cayley, Ilia Filipev, Jeremy A. Davis, Vincent de Rover, Dean Cutajar, Enbang Li, Bradley M. Oborn, Marco Petasecca, Anatoly Rosenfeld, Michael L. F. Lerch

**Affiliations:** ^1^ Centre for Medical Radiation Physics University of Wollongong Wollongong NSW 2522 Australia

**Keywords:** electronic dosimetry, medical radiation physics, radiation medicine, radiation therapy materials science and engineering, silicon dosimeters, solid state detectors

## Abstract

The sophistication and diversity of radiation medicine modalities continue to increase significantly in both cancer imaging and cancer radiation therapy. With over 50% of cancer patients receiving some form of radiation procedure as part of their cancer management plan, the importance and impact of quantitative radiation detector systems for quality assurance in radiation medicine should not be underestimated. There are numerous challenges faced by silicon‐based detector systems used for dosimetry in radiation therapy that are discussed in this review article, especially considering emerging radiation therapy modalities that incorporate ultra‐high irradiation dose‐rates (e.g., MRT, FLASH‐RT, VHEE‐RT), while others utilize mixed radiation fields (e.g., proton‐RT, heavy ion therapy) or diffusion‐based dose delivery techniques (e.g., Alpha DaRT). Such challenges create exciting opportunities for new radiation detector materials. This concise review paper explores the role of silicon in radiation detection applications and highlights the challenges it faces in achieving optimal performance. Additionally, this review discusses new materials that are emerging as strong candidates for the next generation of radiation detectors, emphasizing both the opportunities and challenges associated with these materials.

## Graphical abstract

Silicon has been a cornerstone material of radiation therapy dosimetry for many decades, providing somewhat unchallenged performance. However, there are caveats to its use, requiring complex designs and, often, correction factors to provide accurate measurements. Alternative materials may provide previously unachievable capabilities, ushering in a new generation of radiation detectors suitable for the ever‐changing landscape of radiation therapy.

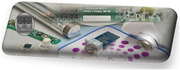

## 1 Introduction

The field of radiation therapy dosimetry requires continual innovation due to the the ever‐changing medical radiation treatment landscape. Emerging modalities create new challenges due to advances in the use of different and changing irradiation environments and also the delivery of such irradiation fields at ultra‐high dose‐rates. The Center for Medical Radiation Physics at the University of Wollongong (CMRP), led by Distinguished Professor Anatoly Rosenfeld,^[^ ^1^ ^]^ has been at the forefront of innovative radiation oncology and medical physics technologies for over 25 years, with a strong focus on the development of solid‐state radiation detectors. Crystalline silicon has been a cornerstone in the construction of these devices, from single diode detectors through to pixelated arrays, with patented manufacturing systems used to produce devices that provide both reference and quality assurance dosimetry in clinical and research facilities worldwide.

Silicon semiconductor detectors have a long history within the field of radiation detection. Since 1970, semiconducting materials have been used, in part, due to their small sensitive volume while providing real‐time readout, high sensitivity, and linear response to absorbed dose.^[^ ^2^ ^]^ One of the most common semiconductor devices used in radiation measurement is the crystalline silicon p‐n junction diode. Operated in either passive or active mode, electron‐hole pairs are created by incident ionizing radiation, and promptly collected at the electrodes due to either the intrinsic potential, or in the case of active devices, by an external bias applied to the device. With an electron‐hole pair creation energy of only 3.6 eV, silicon provides high, yet stable sensitivity. Most silicon detectors are typically read out in real time, by measuring the ionization current produced within the detector by incident radiation. The rate at which electron‐hole pairs are generated determines the magnitude of the current produced within the device and is proportional to the dose‐rate of the irradiation field. The signal can be integrated, to calculate the collected charge and, hence, the total accumulated dose in silicon over the time of irradiation. This opens up opportunities for not only real‐time, but also time‐resolved measurements useful for dynamic treatment modalities and pulsed beams. Well‐established fabrication processes coupled with the ability to produce sensitive volumes of small sizes allow for cost‐effective manufacturing of high spatial resolution 1D and 2D silicon detector arrays.^[^ ^3^ ^]^ As the electron stopping power ratio of silicon to water is almost constant within the energy range of secondary electrons relevant to commonplace megavoltage x‐ray radiotherapy, the corresponding detectors can be considered water‐equivalent for such treatment modalities. Therefore, silicon sensors possess many essential characteristics for dosimetry in radiation therapy. While their known radiation response, relative to human tissue, exhibits limitations due to total accumulated dose effects, irradiation dose‐rate variation, incident radiation field spectral energy variation, and anisotropic radiation therapy treatment delivery, such limitations can, in many cases, be compensated/corrected for and therefore minimized to within acceptable levels for clinical radiation dosimetry.

However, silicon is not a unique material solution to all radiation dosimetry situations. With continual innovation and new, emerging treatment modalities, challenges continue to arise in the radiation detection field of research and development. While the processes governing silicon devices are typically well understood, CMRP continues to expand its repertoire and explore new avenues for radiation detection. As part of this approach, CMRP investigates the use of alternative materials to produce innovative radiation detectors. Often these are in conjunction with silicon, whereas other devices may be entirely silicon free. In this concise review, we first discuss the properties of crystalline silicon detectors, before providing a summary of new and innovative materials used for novel radiation detectors. We explore the mechanisms behind their response, as well as the challenges or opportunities they present. While there are many other exciting materials being investigated globally, such as scintillating polymers and optically stimulated luminescent detectors, the scope of this review is restricted to the detector material research being conducted within CMRP. Finally, we look ahead and present a picture of the future opportunities within the radiation therapy detector material landscape in the context of emerging radiation medicine modalities.

## 2 Challenges for Silicon

Despite the successes of crystalline silicon‐based detectors, there are several inherent challenges that hinder their performance. For a detailed analysis of some of these specific challenges, readers are pointed to dedicated manuscripts produced by CMRP previously.^[^ ^4^, ^5^, ^6^ ^]^ In this section the most important considerations for crystalline silicon in the context of electronic dosimeters for radiation therapy dosimetry are highlighted.

### 2.1 Dose‐Rate Dependence

Silicon detectors are known to exhibit a dependence upon dose‐rate.^[^ ^7^ ^]^ For irradiation environments where the dose‐rate is unchanged, this can be corrected for using a calibration factor for the specific environment. However, in many cases, particularly that of clinical radiotherapy, dose‐rates can vary drastically during delivery, inhibiting the use of a single calibration factor when performing dosimetry and necessitating various diode manufacturing techniques to minimize the dependence. Depending on the type of diode, this can be achieved through radiation priming, altered doping concentrations (electrical resistivity), and/or introduction of specifically designed charge carrier traps and generation‐recombination centers.^[^ ^8^ ^]^

Most clinical beams are delivered in a pulsed manner; therefore, when discussing dose‐rate, it is essential to understand the time structure of radiation beams to derive the dose per pulse (DPP), and instantaneous dose‐rate values, which are critical for the processes happening inside irradiated diodes. With lower dose‐rates typical of current clinical x‐ray modalities, non‐linear dependence on the instantaneous dose‐rate in silicon can be mostly explained by the increasing number of filled generation‐recombination centers with increasing instantaneous dose rate. At higher injection rates when all centres are occupied, the response is independent of the instantaneous dose‐rate, up to the saturation levels when additional electron‐hole recombination is caused by the proximity of electron tracks.

With the emergence of modalities utilizing high and ultra‐high dose‐rates (UHDR) as high as MGy/s, such as x‐ray stereotactic radiotherapy, FLASH‐RT, very high‐energy electron radiotherapy (VHEE‐RT), and Microbeam Radiation Therapy (MRT), dose‐rate independence is an increasingly desirable quality of any detector.^[^ ^9^ ^]^ A diode‐based detector was shown to exhibit significant saturation to a DPP of only 10 cGy/pulse.^[^ ^10^ ^]^ More recent studies, however, have suggested that biased silicon p‐i‐n diodes with thin epitaxial sensitive volumes display strong potential in UHDR electron beam monitoring, with a constant response up to 10.22 Gy/pulse (instantaneous dose rate up to 2.5 × 10^6^ Gy/s with 4 μs 1 Hz pulses).^[^ ^11^ ^]^

### 2.2 Energy Dependence

Within any clinical setting, the primary purpose of radiation dosimetry is to determine the absorbed dose to human tissue. In most cases, measurements are performed in water or water‐equivalent materials due to simplicity and compliance with dosimetry standards and regulatory guidelines. The interaction of ionizing radiation with matter (water), and thus the resulting energy deposition, varies depending on the type and energy of the radiation. Therefore, an ideal dosimeter would be made of the same material as the surrounding water to ensure similar interaction mechanisms and dependencies for the incoming radiation.

**Figure 1:**
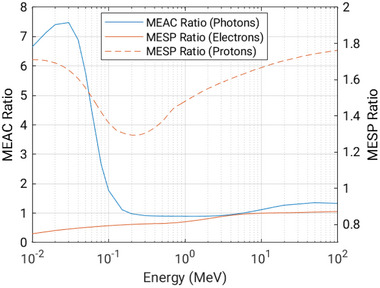
The ratio of mass energy absorption coefficients (MEAC), and mass electronic stopping powers (MESP), for silicon relative to water.

Silicon‐based dosimeters interact with ionizing radiation differently, which can be explained for photon, electron, and proton beams from silicon‐to‐water ratios for mass electronic stopping powers and mass energy absorption coefficients as a function of energy, shown in **Figure** **1**.

If the dose is delivered by a field of electrons or protons, then the dose to water can be calculated using the ratio of mass electronic stopping powers averaged over the energy spectrum, provided the detector is small relative to the field, as well as to the range of charged particles, ensuring charged particle equilibrium. From Figure 1 it can be seen that for typical electron energy ranges used in radiation therapy, the ratio is relatively constant and therefore silicon can be considered water‐equivalent or energy‐independent. For protons, the ratio varies considerably with energy, hence, silicon is not considered water equivalent across these energies.

For a photon field, however, due to which production of secondary electrons occurs within the silicon, though not in the surrounding water the ratio of mass attenuation coefficients must also be considered. In the kilovoltage range, due to the higher atomic number of silicon compared to water, and the resulting increased cross‐section for the photoelectric effect, silicon‐based dosimeters are more sensitive to photons and exhibit strong energy dependence. In the megavoltage range, the response between silicon and water is more consistent and so energy dependence is not observed strongly for these energies.^[^ ^4^, ^12^, ^13^, ^14^ ^]^

### 2.3 Radiation Field Effects

Radiation therapy extends to charged and uncharged particle based therapies (e.g. proton therapy, carbon ion, boron neutron capture therapy, and fast neutron therapy), with the most common being proton therapy. In these, therapies the local radiation field environment can change markedly (e.g., Bragg peak region), and is commonly referred to as a mixed radiation field. This mixed radiation field can give rise to a significant therapeutic advantage compared to photon therapies, driven by a radiobiological response in human tissues, but is very challenging to measure an equivalent quantitative dose response using silicon detectors.^[^ ^15^ ^]^ These quantitative dose measurements are based upon microdosimetry, which is the measurement of the stochastic energy deposition by charged particles in extremely small volumes. Measurements are recorded event‐by‐event in micron‐sized sensitive volumes, similar in size to that of a human cell.^[^ ^16^ ^]^

For these types of therapies, radiation detectors capable of microdosimetric measurements are essential.^[^ ^17^ ^]^ The radiobiological related observations reported in the literature from such microdosimetry measurements, and their biochemical associated implications in such radiation environments are very important for both non‐cancerous and cancerous tissues. The efficacy of these therapies is deduced by this dosimetry technique.

A 2D array of silicon volumes is used to mimic the cellular dimensions. Accurate results require precise knowledge of the Si sensitive volumes, as well as ideally, no cross‐talk between volumes. Spectroscopic grade (5–10 kΩcm) n‐type silicon is typically used to formulate the sensitive volume arrays and as such they are susceptible to bulk and surface interface related radiation damage effects induced in and around the sensitive volumes by the mixed radiation field environment, which is a challenge that needs to be addressed when interpreting the microdosimetric data.

**Figure 2:**
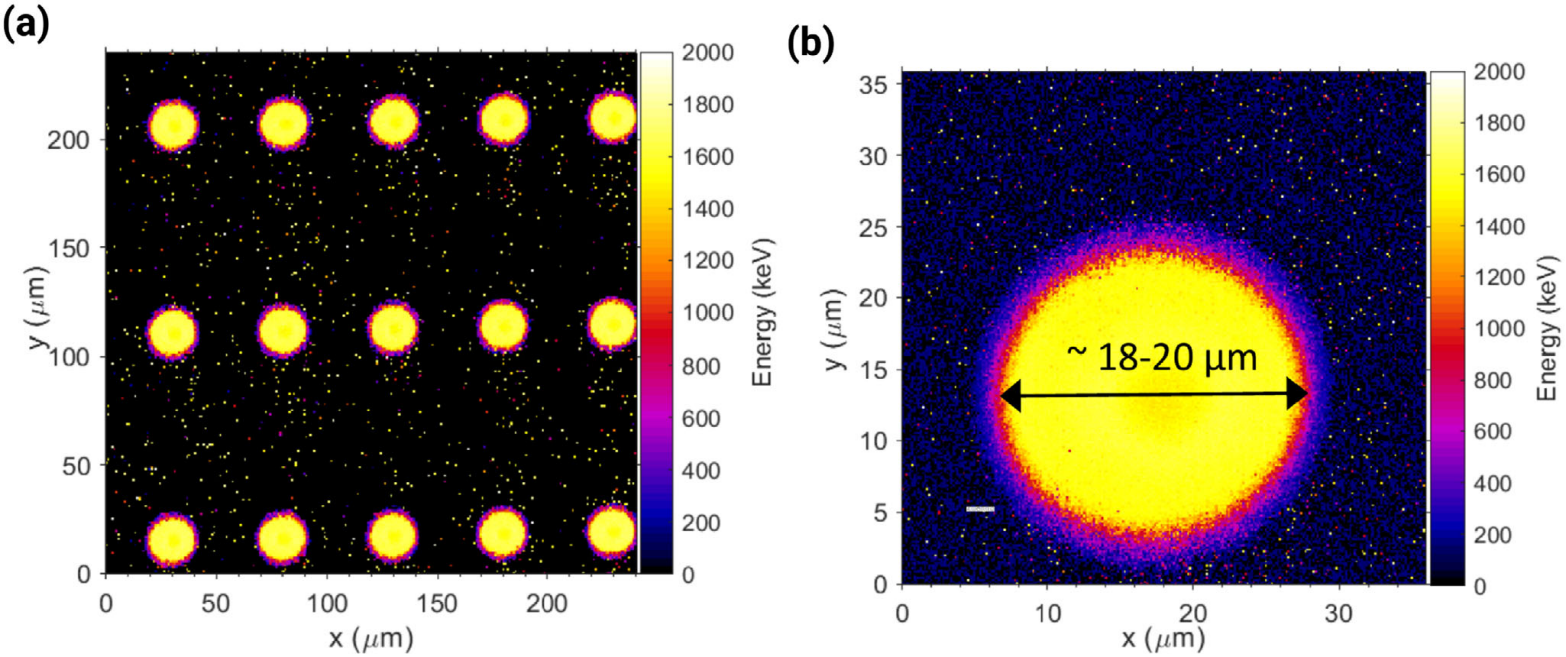
a) The response of a 10 μm thick mushroom detector array to a 5.5 MeV He^2+^ ion beam. b) Cropped mean energy map of a single SV within the array, approximately 18–20 μm in diameter. a) and b) reproduced from^[^ ^18^ ^]^ under Creative Commons licence, 2019, IOPScience.

CMRP introduced the concept of Silicon On Insulator (SOI) detectors for microdosimetry which are based on micron‐sized silicon cylindrical sensitive volume (SV) arrays to achieve the required mimicking of human cell size.^[^ ^16^ ^]^ These detectors were fabricated at SINTEF MiNaLab, Norway using 3D detector technology. This allows production of well‐defined 3D SVs within the detector by using the deep reactive ion etching process. This process utilises a sequence of alternate etching and passivation processes, creating high aspect ratio side walls of cylindrical shape, followed by gas doping of p+ on this wall, n+ as the core electrode and a p‐Si active layer with an SV thickness of 2, 5, and 10 μm.^[^ ^19^ ^]^ Such SOI detectors for microdosimetry are well known as ‘mushrooms’. This innovative leap in detector manufacturing technology allows highly precise measurements, with markedly absent cross‐talk between SVs. **Figure** **2**a) displays the median energy map of measurements undertaken with a 10 μm thick mushroom detector during exposure to a 5.5 MeV He^2+^ ion beam. The desirable qualities for microdosimetry are clearly evident, with Figure 2b) providing the measurements from a single SV, with a diameter of approximately 18–20 μm.

While the above results signify important advancements for microdosimetric measurements, the use of silicon does present challenges due to build‐up of charge within the SiO_2_ field layer, and event‐pulse pile‐up effects when exposed to high dose‐rate irradiation fields. When SOI microdosimeters were exposed to 24 MeV carbon ions (corresponding to 1 MeV neutron equivalent fluence, approximately 2 × 10^15^ cm^−2^), the effect of built‐up charge due to previous gamma exposure of 10 Mrad(Si) caused parasitic low energy events ^[^ ^20^ ^]^. High dose‐rates will cause charge pile up within the SOI microdosimeter, rendering these devices unsuitable for emerging modalities that incorporate ultra‐high dose‐rates such as FLASH radiotherapy. However, this limitation exists not only for SOI devices, but for any available microdosimeter including tissue equivalent proportional counters, known as the gold standard for microdosimetry.^[^ ^21^ ^]^

### 2.4 Radiation Hardness

**Figure 3:**
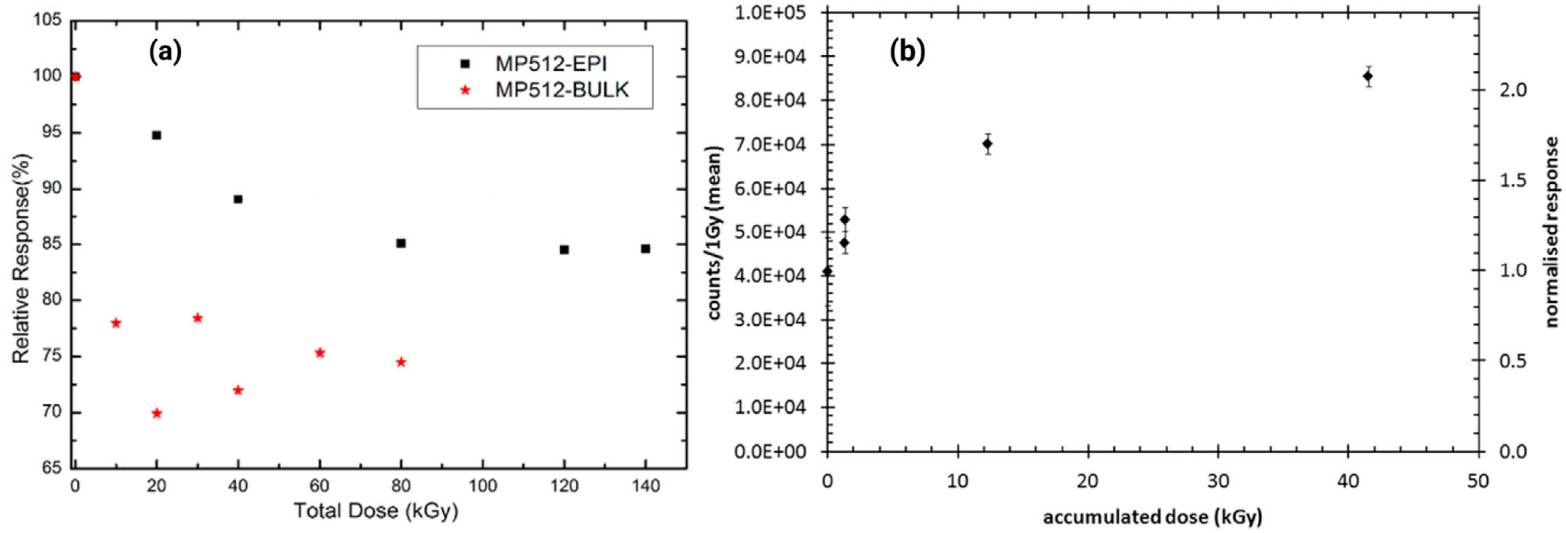
a) Sensitivity degradation with accumulated dose for the MagicPlate‐512 (MP512) monolithic array detectors with ion‐implanted diodes fabricated on a p‐type substrate (epitaxial and bulk designs). Used with permission from Springer Nature BV from ^[^ ^24^ ^]^; permission conveyed through Copyright Clearance Center, Inc. 2024 b) Sensitivity change with accumulated dose for Magic Plate detector ‐ array of epitaxial silicon diodes grown on p‐type substrate. The surface damage effects cause positive charge build‐up at the SiO_2_ field oxide layer leading to the apparent increase in the sensitivity with dose. Reproduced from ^[^ ^25^ ^]^ with permission from John Wiley and Sons, 2012.

The sensitivity, and consequently the response, of silicon detectors is known to vary with accumulated dose as a result of a combination of bulk and surface damage, typically leading to a degradation in detector response, shown in **Figure** **3**, as well as increased leakage current (for biased detectors), and possible decrease in the signal‐to‐noise ratio.^[^ ^22^, ^23^ ^]^

In clinical radiotherapy settings, radiation hardness typically refers to a detector's ability to maintain stable performance without requiring recalibration more than once or twice per year under routine use ‐ corresponding to degradation levels of about 1% per kGy or less.^[^ ^26^ ^]^ However, with the emergence of hypofractionated treatment regimens and new spatially fractionated techniques such as MRT, detectors are often exposed to significantly higher doses during each verification procedure. MRT, for instance, can deliver hundreds, even thousands, of Gy in fractions of a second.^[^ ^27^ ^]^ Stability of the detector response over a much larger range of absorbed doses is a highly desirable trait of detectors employed in this area of research.

Silicon detectors are typically stabiliZed by reducing the minority charge carrier lifetime through the introduction of suitable generation‐recombination centers into the crystal lattice. This can be achieved via preirradiation (also known as priming) and/or by doping with platinum. The required priming dose varies between devices but is generally above 10–20 kGy and can reach up to 80 kGy, after which the detector response tends to plateau.^[^ ^28^ ^]^ At this point, the detector is considered stable and suitable for continued use. Sensitivity stability with respect to accumulated dose can also be achieved by controlling the sensitive volume of the diodes during fabrication by ensuring that the diode dimensions are smaller than the expected diffusion length of minority charge carriers. This can be realised through the use of thin epitaxial layers grown on low‐resistivity substrates and the lateral introduction of guard rings.

### 2.5 Response Anisotropy

Silicon has a relatively high atomic number (Z) compared to water and commonly used detector packaging materials such as plastic and printed circuit boards made of polyimide or FR‐4, a fiberglass impregnated resin board. Additionally, the sensitive volumes are often manufactured in a planar geometry. As a result, most silicon detectors exhibit intrinsic asymmetry in their sensitive layers due to the presence of surrounding materials with differing atomic compositions. This leads to asymmetric perturbation of the radiation field dependent upon the angle of incidence of the beam. In monolithic diode arrays (the fabrication of multiple diodes on a single substrate, i.e., by ion implantation), the presence of silicon between the diodes (and above or below them in the case of epitaxial technology) adds to the radiation field perturbation significantly.^[^ ^3^, ^29^, ^30^ ^]^

Commonly referred to as an angular dependence, this effect can be minimized or compensated for by manipulating either the sensitive volume and charge collection geometry or the geometry of the surrounding packaging. The former approach aims to achieve isotropic charge collection by carefully engineering the p‐n junction. Methods by which this can be achieved include manufacturing a spherically symmetric diode^[^ ^31^, ^32^, ^33^ ^]^ or an edgeless planar diode configuration.^[^ ^34^, ^35^, ^36^, ^37^ ^]^ In terms of detector packaging, geometric imbalance can be addressed by strategies such as placing two substrates back‐to‐back, incorporating high‐Z materials to equalize the response across beam directions,^[^ ^38^, ^39^ ^]^ or by using CMRP developed drop‐in manufacturing technology.^[^ ^40^ ^]^

## 3 New Materials in Radiation Detection

Though the properties of crystalline silicon, discussed briefly above, are well understood and have allowed for the development of numerous detector technologies, other materials may offer benefits that enhance the ability to quantify the radiation fields of new, increasingly demanding radiotherapy modalities. Many of these materials offer solutions to challenges faced by silicon, presenting an opportunity to design novel devices. However, in isolation, they may not present a single solution. Below, we present some of the materials currently investigated by CMRP for the development of entirely new detectors that may usher in a new generation of radiation detection capabilities.

### 3.1 Hydrogenated Amorphous Silicon

CMRP is a member of an international collaboration named HASPIDE (Hydrogenated Amorphous Silicon PIxel DEtectors), funded by the Italian Instituto Nazionale di Fisica Nucleare and established to pursue the development of a‐Si:H detectors built on flexible substrates for real‐time, and in vivo dosimetry. In many European countries, in vivo dosimetry is now mandatory according to the Medical Exposure Directive.^[^ ^41^ ^]^ Eminent studies have recognized the technological gap between regulations and commercially available radiation detectors that are suitable for in vivo dosimetry in several radiotherapy modalities.^[^ ^42^ ^]^ Hydrogenated amorphous silicon (a‐Si:H), first developed in 1969,^[^ ^43^ ^]^ is a disordered semiconductor material that has since found widespread application in electronics, photovoltaics, and radiation detection.^[^ ^44^, ^45^ ^]^

The predominant method for fabrication of a‐Si:H substrates is plasma enhanced chemical vapor deposition (PECVD), utilizing a gas mixture of silane (SiH_4_) and hydrogen at temperatures ranging from 180 to $250^{\circ }C$.^[^ ^46^ ^]^ This low temperature processing capability facilitates direct deposition onto flexible substrates, such as polyimide.

Typically, the device architecture for radiation detection employs an n‐i‐p diode structure. This comprises an n‐doped a‐Si:H layer deposited onto a metal stack ‐ typically aluminium and chromium ‐ that serves as the backside contact. Above this, an intrinsic a‐Si:H layer functions as the active region where incident radiation induces ionization, leading to the generation of e‐h pairs. These charge carriers are subsequently separated and collected by an electric field established through external biasing.^[^ ^47^ ^]^ The structure is completed by the deposition of a p‐doped a‐Si:H layer and a top contact, which may consist of either a metal layer or indium tin oxide, to facilitate efficient charge extraction.

The challenge when developing ionizing radiation direct detection sensors (converting the energy deposited by radiation directly into the substrate into charge) based on a‐Si:H is the requirement to fabricate relatively thicker substrates when compared to traditional thin film technology developed for solar power harvesting. The substrates investigated by the HASPIDE project range from 2.5 to 30 μm of intrinsic a‐Si:H, presenting a significant challenge as efficient charge extraction is of paramount importance. Additionally, deposition on flexible substrates without delamination or impacting upon the bulk material integrity requires a refined fabrication procedure. The key elements for controlling the quality of the substrate are the concentration and pressure of the silane gas during the deposition, as well as the growth rate and temperature gradients. These parameters control the concentration and distribution of Si‐H/H_2_ bonding, responsible for the satisfaction of the defects created by the amorphous silicon matrix. The accumulation of unsatisfied Si bonds generates defects in thick substrates in the forms of traps and thermal donors which are detrimental to maintaining an optimal charge collection efficiency.

A considerable effort has been made to develop a simulation model based on the theory of deep level pool defects and implemented in SYNOPSYS TCAD. The study has demonstrated how the concentration of defects in the bandgap of an n‐i‐p a‐Si:H substrate, modulated by the presence of unsatisfied Si bonds and Hydrogen atoms, is able to mimic the shape and variation of the leakage current as a function of the applied reverse bias and also as a function of the temperature.^[^ ^48^, ^49^ ^]^ These results have been confirmed experimentally by electrical characterizations, Raman spectroscopy, photoemission, and inverse photoemission techniques applied to samples fabricated on both thick and thin substrates.^[^ ^50^ ^]^ This study also showed the variations in substrate quality associated with a‐Si:H grown on a crystalline substrate or on a plastic substrate such as polyimide. Bandgap and low‐range disorder affect the sensitivity of the final device with polyimide‐based diodes showing a larger bandgap (1.9 eV) in respect to the same substrate grown on crystalline silicon (1.7 eV).

**Figure 4:**
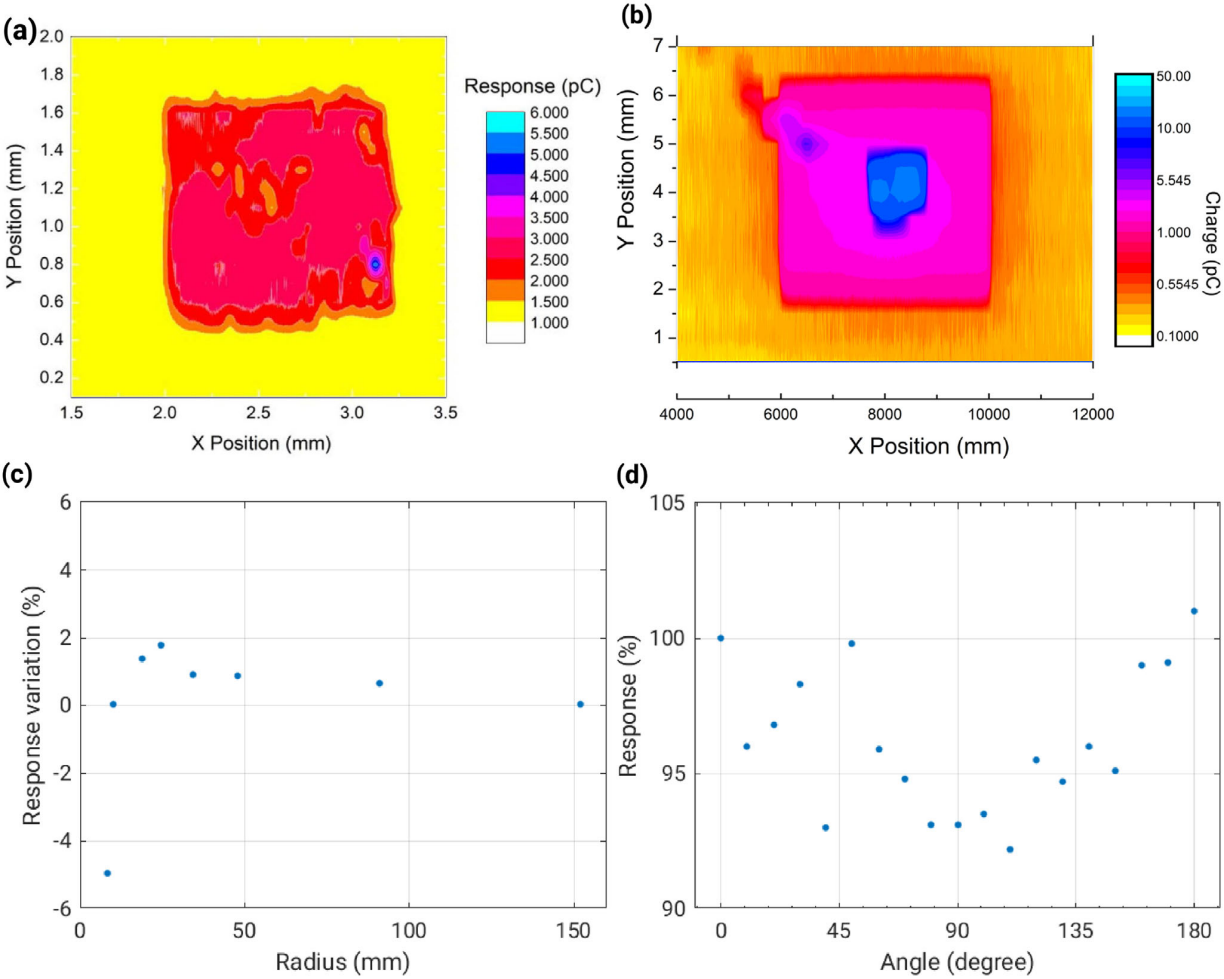
a) The results of Synchrotron X‐ray Beam Induced Current (XBIC) measurements confirming the high and more evenly distributed sensitivity of the CSC device as opposed to n‐i‐p devices. b) XBIC measurements for n‐i‐p detectors. c) a‐Si:H detectors were subjected to bending, showing a promisingly constant response with bending radii as low as 7 mm. d) Through a full 180° rotation of the linac gantry, a‐Si:H detectors exhibited a low dependence upon the angle of incidence of radiation.

The HASPIDE project has also carried out an extensive investigation on contacts and interface effects. Charge‐selective contact (CSC) devices represent an alternative to the conventional n‐i‐p configuration, replacing the n‐type and p‐type layers with electron‐selective and hole‐selective contact materials, respectively. In the present implementation, titanium dioxide (TiO_2_) serves as the electron‐selective layer, while molybdenum oxide (MoOx) is employed for hole selectivity. a‐Si:H CSC devices typically comprise a trilayer stack: a thin metal‐oxide layer with a low activation energy (e.g., TiO_2_), a central thick intrinsic a‐Si:H layer, and a final thin metal‐oxide layer with a high activation energy (e.g., MoOx or tungsten oxide, WOx).^[^ ^45^ ^]^ The contact type defines a trade‐off between low leakage currents and high sensitivity: shown in **Figure** **4**a,b, it has been proved that CSC provides a higher and more evenly distributed sensitivity to ionizing radiation (from 40 to 120 pC/cGy. mm^2^ ) in respect to n‐i‐p diodes (0.5 to 20 pC/cGy. mm^2^ ),^[^ ^51^ ^]^ while higher leakage currents are recorded for CSC structures in respect to n‐i‐p devices. In clinical dosimetry, a passive polarization of the detector is preferable for stability of the response and safety of the patient, rendering the CSC an optimal solution for medical applications.

Radiation hardness is also an important aspect of characterization of radiation sensors used for dosimetry where stability of the response is critical. Thick a‐Si:H detectors have been proven to withstand very large doses (up to 800 Mrad) of gamma and up to 1016 cm^−2^ neutron irradiations, displaying stability within a few percent after an initial, strong variation of the responsivity.^[^ ^52^ ^]^ The causes of the preliminary reduction of the sensitivity have been investigated using soft x‐ray spectroscopy, which highlighted a strong reduction of the concentration of Si‐H bonds with a considerable increase of the short range disorder of the substrate.^[^ ^53^ ^]^

The a‐Si:H detectors have been tested as dosimeters in 6MV linear accelerators for phantom quality assurance, proving to be a reliable device with some peculiar advantages over commercially available detector technologies: they proved to be angularly independent, with a minimal response variation during a +/‐180 degree rotation of the beam around the sensor, which can be observed in Figure 8d). They proved to be tissue equivalent at this range of energies with no perturbation detectable relative to ionization chambers when measuring percentage depth‐dose distributions and field factor variations. They also proved to be insensitive to bending, within 2%, shown in Figure 8c) with a radius of curvature from 100 to 7 mm and conserved their responsiveness after several bending and relaxation cycles.^[^ ^54^ ^]^

**Figure 5:**
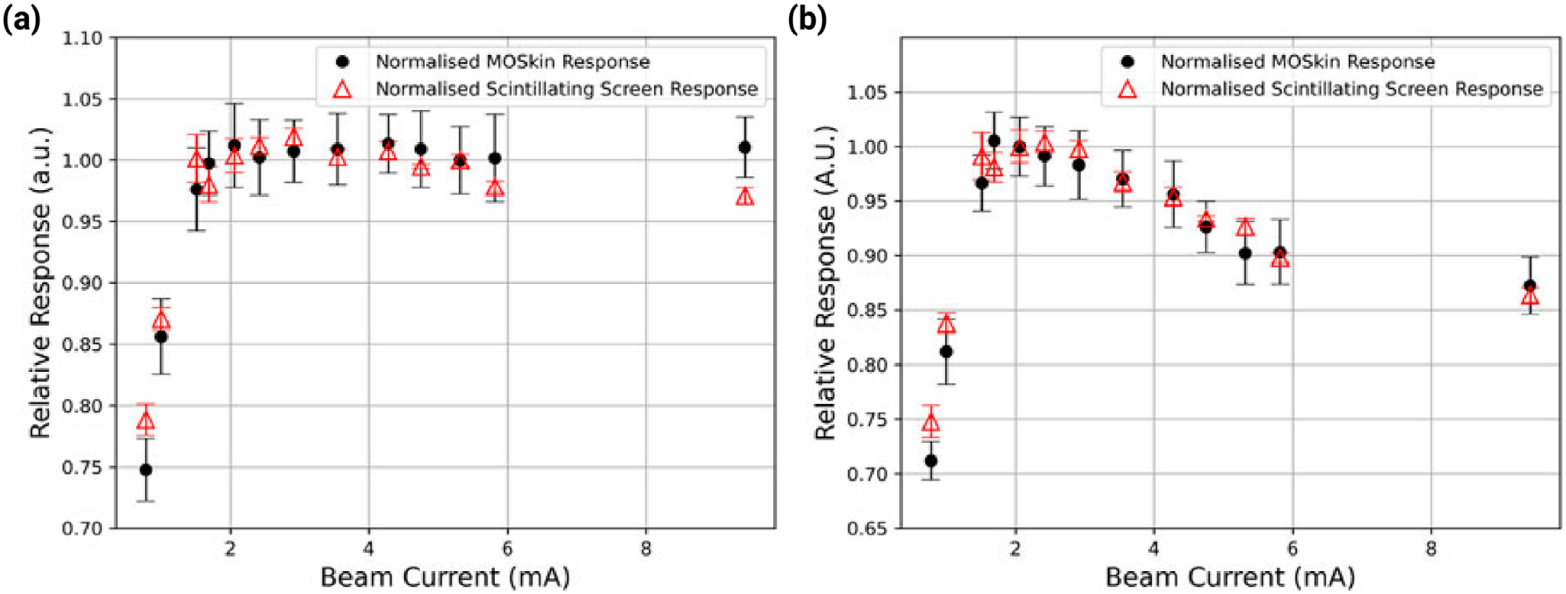
a,b) UHDR MOSkin results for two MOS*kin* detectors positioned at different locations within a 100 MeV electron field, with a Gaussian spatial distribution. Reproduced under Creative Commons license, 2024, Frontiers from.^[^ ^56^ ^]^

**Figure 6:**
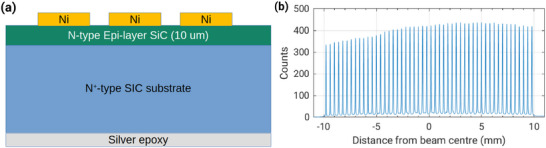
a) Cross‐section of the SiC detector used in preliminary CMRP investigations.^[^ ^87^ ^]^ b) The full 50 microbeam field resolved by the SiC detector scanning laterally through the beam.

**Figure 7:**
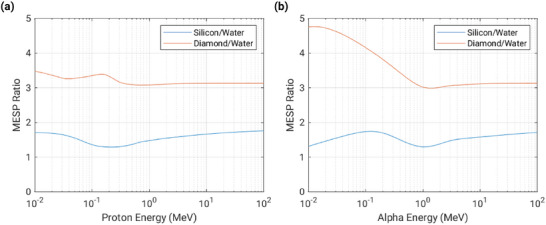
Mass electronic stopping power ratios of diamond relative to water, and silicon relative to water, for (a) protons and (b) alpha particles.

**Figure 8:**
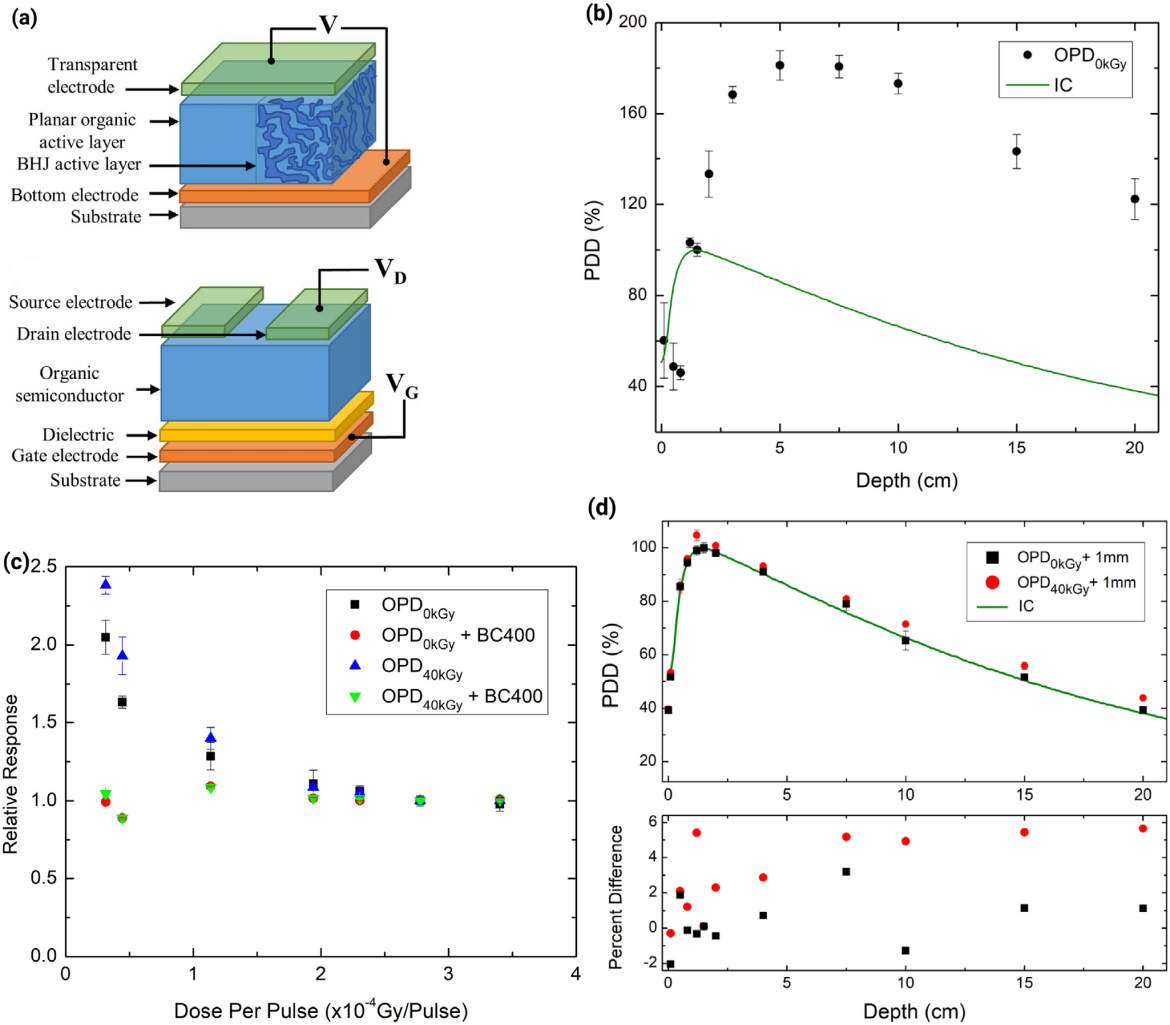
a) Example of prominent multilayer organic structures, (top) an organic BHJ diode, (bottom) an organic field effect transistor. Reproduced with permission from IOP Publishing Ltd from Ref. [110]; permission conveyed through Copyright Clearance Center, Inc. 2021 (b) The overresponse of an organic diode used as a direct detector. c) The dose‐rate response of the same organic diode in both direct and indirect detection modes. d) The organic diode correctly reproduced accurate percentage depth dose measurements when coupled with an organic scintillator. b–d) reproduced with permission from Ref. [121]; permission conveyed through Copyright Clearance Center, Inc, 2021, Wiley.

Testing has also been conducted for suitability as a radiation dosimeter for MRT quality assurance. MRT is a pre‐clinical modality that adopts high fluxes of synchrotron light (providing dose‐rates up to 10kGy/s) with a high degree of spatial fractionation (the beam is comb collimated by 50 μm wide apertures spaced 400 μm apart). Two studies on n‐i‐p and CSC devices, carried out at the Australian Synchrotron, have shown an agreement within 3% of the percentage depth dose recorded by the CSC samples and a PTW microDiamond commercial detector. Although the n‐i‐p devices showed a better radiation hardness in comparison to CSC diodes, the late configuration of contacts has a smaller impact on energy dependence of the response, reproducing the Peak to Valley Ratio (PVDR) as a function of depth in a solid water phantom in excellent agreement with microDiamond and Monte Carlo simulations.^[^ ^55^ ^]^

Research activity on a‐Si:H diodes continues with the aim of developing specialized geometries to create pixel arrays on flexible kapton substrates. This option will allow the investigation of the sensor as a tissue equivalent in‐vivo dosimeter for skin dose mapping or development of pixelated active membranes for online beam monitoring.

### 3.2 Silicon Dioxide

Silicon dioxide is commonly used as the dielectric gate oxide in metal oxide semiconductor field effect transistors (MOSFETs). Though these devices are otherwise silicon‐based, the mechanism by which MOSFETs produce a response to ionizing radiation differs significantly from that of conventional silicon‐based detectors.

As a four‐terminal device, MOSFETs are commonly used to switch a current between the source and drain terminals by applying a voltage to the gate. This voltage, known as the threshold voltage (*V*_*th*_), attracts minority carriers in the substrate creating a conduction channel between the source and drain. A p‐channel MOSFET (named for the sign of the minority carriers that form the channel) is manufactured upon an n‐type silicon substrate, and the reverse is true for n‐channel devices. The phenomena upon which MOSFET detectors rely upon, is the creation of electron‐hole pairs within the gate oxide as the device is exposed to ionizing radiation. Mostly used as active devices, an external bias causes the holes to drift towards the SiO_2_ interface, where they become permanently trapped within interface defect centers. This trapping of charge causes a change in the conduction channel through Coulombic repulsion. Hence, to continue passing a constant current between the source and drain, an increased *V*_*th*_ is required as the device is further exposed to radiation. This shift in *V*_*th*_, Δ*V*, is linearly proportional to absorbed dose and is a measure of the sensitivity of the device, expressed as Δ*V*/1cGy. As the holes become trapped permanently, MOSFETs produce integrating dosimeters, hence, their response is proportional to total dose, rather than to dose‐rate as for other semiconductor‐based detectors. This can be particularly useful in low dose‐rate environments where other detectors that rely upon ionization current as the measurable response may not be sensitive enough to provide results with high signal to noise ratios. While MOSFETs are commonly manufactured on the nanometre scale for commercial electronics, as a radiation detector the response of the device is proportional to the square of the thickness of the oxide. This property can be used to control the sensitivity of the device as required for different radiation fields. The micrometer‐scale physical dimensions of the radiation detection sensitive volume of MOSFETs makes them ideal candidates for small‐field dosimetry and quality assurance of related radiation treatments.^[^ ^57^ ^]^

The CMRP designed MOS*kin* is manufactured with a gate oxide thickness ranging from 0.55 to 1 μm. Coupling patented packaging design with such a thin sensitive volume allows the MOS*kin* to exhibit the water equivalent depth required to accurately measure the skin dose during clinical radiotherapy,^[^ ^58^, ^59^, ^60^, ^61^ ^]^ defined as the radio‐sensitive basal layer residing 70 μm below the surface.^[^ ^62^ ^]^ With the previously discussed requirements for in vivo dosimetry in many European countries, measurements of the skin dose, as well as internal measurements^[^ ^63^, ^64^, ^65^ ^]^ become increasingly important metrics for all radiotherapy modalities.^[^ ^66^, ^67^ ^]^

The combination of these properties allowed the innovative use of the MOS*kin* to measure the skin‐dose during novel synchrotron computed tomography (CT) investigations at the Imaging and Medical Beamline at the Australian Synchrotron. Having previously been tested within clinical CT environments,^[^ ^68^ ^]^ not only was the MOS*kin* able to produce a response in the extremely low dose‐rate synchrotron imaging environment, it also confirmed the presence of a threefold increase in dose at the entrance, relative to the center of the breast.^[^ ^69^ ^]^ These results were then confirmed with Geant4 Monte Carlo simulations.

MOS*kin*s have also been tested within the mixed fields of diffusing alpha‐emitters radiation therapy (Alpha DaRT) and targeted alpha‐particle therapy, composed of α particles, β particles, and γ rays. The high linear energy transfer (LET) α particles place an important consideration upon the use of in vivo dosimetry, as the dose is deposited over a very short range and could have catastrophic results if unintended or unaccounted for. A linear response was observed up to an absorbed dose of 25 Gy, with the sensitivity of the detectors controllable by external applied bias between 15 and 60 V,^[^ ^70^ ^]^ demonstrating these devices can be optimized for these extreme fields.

Like silicon, the gate oxide of MOSFET devices is not water equivalent at all energies and the detector response is, therefore, dependent upon the energy and particle type of the incident radiation field. Hence, as is the case for most detectors, a calibration against reference dosimetry is required to establish the sensitivity of the device, used to calculate the value of dose to water. Unlike silicon, however, MOSFETs do not exhibit a dose‐rate dependence for an unchanged beam quality,^[^ ^67^, ^71^, ^72^ ^]^ making them particularly useful for emerging radiotherapy modalities such as FLASH radiotherapy. Investigations have been made into the dose‐rate independence of the MOS*kin* for VHEE‐RT, which commonly utilises ultra‐high dose‐rates, many orders of magnitude greater than that of the 40 Gy/s threshold upon which the FLASH regime is defined.^[^ ^73^ ^]^ At an energy of 100 MeV, the PEER (Pulsed Energetic Electrons for Research) beamline at the Australian Synchrotron was used to irradiate MOS*kin*s at varied accelerator beam currents corresponding to exposure times of less than 400 ns, with average and instantaneous dose‐rates as high as 1.28 × 10^7^ Gy/s and 2.55 × 10^8^ Gy/s, respectively. The detector showed a flat response across all but the lowest dose‐rates.^[^ ^56^ ^]^ However, the response of a 2D scintillator showed a change in the spatial distribution of the beam (both the shape of the Gaussian distribution, as well as the central positioning), and a loss of charge from the linac. When normalized results, shown in **Figure** **5** for the MOS*kin* and scintillator were compared, the MOS*kin* was shown to be responding proportionally to the electron fluence, and not exhibiting a dose‐rate dependence, in agreement with expectation.

The unfavorable consequence of any integrating dosimeter, where MOSFET technology is no exception, is that a saturation will eventually occur, effectively limiting the lifetime of the detector. The reversal of this effect has been shown feasible for the MOS*kin* in a previous study.^[^ ^74^ ^]^ CMRP investigated the use of direct, and pulsed current annealing, two methods for injecting hot charged carriers into the gate oxide, enabling the repeat use of a previously saturated detector. The study also showed that the initial *V*_*th*_ of an unirradiated detector can be shifted lower, effectively increasing the linear range of the detector. The reuse of MOS*kin* dosimeters is not only an important step forward for sustainability, an issue that faces all industries including radiation therapy, it also solves the most prominent challenge for MOSFET dosimetry. Currently, the sensitivity must be recalibrated after this process, though ongoing efforts are progressing toward further understanding of the processes required to predict the response post‐annealing.

### 3.3 Silicon Carbide

Silicon carbide (SiC) detectors have emerged to help address some of the limitations associated with silicon detectors, particularly when looking into harsh radiation environments. There are several different polytypes available for SiC detectors, but due to its stability and wider bandgap, 4H‐SiC is the most common polytype used for SiC radiation detectors. The bandgap for 4H‐SiC is 3.27 eV, which is almost three times greater than that of silicon detectors at 1.12eV.^[^ ^75^, ^76^ ^]^ As a result of the wider bandgap, SiC detectors experience lower leakage current and noise,^[^ ^77^ ^]^ as well as being more resistant to radiation damage for gamma‐rays.^[^ ^76^ ^]^ They also exhibit negligible effects on alpha particle response (when the reverse bias is increased) for doses up to 5 MGy ^[^ ^78^ ^]^ and minimal change in reverse current‐voltage characteristics for doses up to at least 400 kGy.^[^ ^79^ ^]^ However, a consequence of the wider bandgap is that SiC detectors are less sensitive to radiation than silicon detectors. The electron and hole mobility for SiC, 800 and 115  cm^2^ V^−1^s^−1^, respectively, are lower than that of silicon where the electron mobility is 1350  cm^2^ V^−1^s^−1^ and hole mobility is 480  cm^2^ V^−1^s^−1^.^[^ ^75^, ^76^ ^]^ Effects of the lower mobilities can be compensated for, however, as the internal electric field that a SiC detector can withstand is 8 to 10 times greater than for silicon detectors.^[^ ^80^ ^]^ Consequently, the saturated electron drift velocity for SiC, 2 × 10^7^ cms^−1^, is higher than that of Si at 0.8 × 10^7^ cms^−1^. SiC exhibits a response closer to that of tissue, due to a smaller difference between its effective atomic number (Z_eff_ = 10) and that of soft tissue (Z_eff_ = 7.42), when compared to silicon (Z = 14). While other materials may offer further improvements in this respect, a reduced dependence upon energy is, of course, a desirable quality. Strong performance has been observed in ultra‐high dose‐rate environments; dose‐rate independence was confirmed within fields delivered as high as 4 MGy/s during pulsed electron beam experiments.^[^ ^81^ ^]^

Detectors constructed of SiC suffer from limitations as a result of the large number of lattice defects within the material, as opposed to purely silicon devices. This is particularly problematic, as these defects can act as charge trapping centers, the presence of which reduces the lifetime and mobility of charge carriers, negatively impacting the charge collection efficiency and overall detector performance.^[^ ^82^, ^83^, ^84^ ^]^ Compared to silicon detectors, the manufacturing process of SiC detectors is more complex, requiring high temperatures in excess of $1000^{\circ }C$ to achieve high‐quality substrates and epitaxial layers.^[^ ^75^, ^85^, ^86^ ^]^

CMRP has conducted preliminary investigations utilizing high dose‐rate synchrotron radiation fields, with a view towards their use within MRT research. For a SiC detector to be used as a dosimeter in MRT, it should provide adequate spatial resolution, possess a large dynamic range, and allow real‐time measurements [14]. An epitaxial silicon carbide detector with nickel (Ni), Schottky type contacts, was produced and manufactured by SINTEF. **Figure** **6**a, shows the structure of the device where the epitaxial layer was manufactured with a thickness of 10 μm.

Within a high dose‐rate synchrotron x‐ray field at the Imaging and Medical Beamline of the Australian Synchrotron, the detector was scanned laterally through a microbeam field in edge‐on configuration. Figure 6b shows that the SiC detector achieved adequate spatial resolution, resolving all 50 microbeams that passed over the detector's sensitive volume. The average FWHM of each microbeam was calculated to be (54 ± 1) μm.^[^ ^88^ ^]^ Currently, it is thought that the reason for the slightly larger microbeam width may be due to the dose ‘blurring’ over the sensitive volume, or an angular dependence as a result of possible rotational misalignment. Although investigations are ongoing, the performance of the SiC detector within this initial work demonstrated suitability for microbeam radiation fields.

### 3.4 Diamond Detectors

Currently, diamond‐based detectors are being utilized in a wide variety of applications from high‐energy physics, beamline and reactor monitoring, radioprotection in space and other harsh radiation environments to quality assurance in medical dosimetry. Diamond‐based radiation detectors present a compelling alternative to silicon for specific applications and/or harsh radiation environments in which the advantages of diamond (see **Table** **1**), such as radiation hardness, fast response, low noise and tissue equivalence, justify their significantly higher cost.

**Table 1:** Comparison of properties between Diamond and Silicon. Data adapted from Ref. [89]

Property	Diamond	Silicon
Density (g cm^−3^)	3.5	2.33
Band‐gap (eV)	5.47	1.12
Dielectric constant	5.7	11.7
Resistivity (Ω)	>10^12^	2.23 × 10^5^
Breakdown voltage (V cm^−1^)	10^7^	3 × 10^5^ (pn)
Electron mobility, µ_*e*_ (cm^2^V^−1^s^−1^)	2400	1350
Hole mobility, µ_*h*_ (cm^2^V^−1^s^−1^)	2100	480
e‐h pair creation energy (eV)	13	3.6
Thermal conductivity (W m^−1^ K^−1^)	∼2000	150

Diamond‐based radiation detectors have been investigated since the mid‐1900s,^[^ ^90^ ^]^ with the earliest prototypes primarily fabricated from natural diamond.^[^ ^91^ ^]^ However, over the past two decades, the development of these detectors has shifted towards the use of synthetic single‐crystal and polycrystalline diamond.^[^ ^89^ ^]^ The most widely adopted method for producing high‐quality, electronic‐grade diamond is chemical vapor deposition (CVD). Advances in fabrication techniques have enabled the production of diamond with minimal impurities, typically nitrogen at levels of a few parts‐per‐billion.^[^ ^89^ ^]^

The use of electronic‐grade diamond in detector construction significantly mitigates issues associated with deep and low‐level traps caused by impurities, which were prevalent in early natural diamond‐based prototypes.^[^ ^89^ ^]^ Although the current state‐of‐the‐art in CVD‐based diamond production can yield consistent, high‐quality diamond samples, there remains room for improvement, particularly in the size of crystals that can be grown. For comparison, CVD‐based manufacturing typically produces diamond samples with a surface area of approximately 5 × 5  mm^2^ , whereas silicon fabrication techniques, particularly the Magnetic Czochralski method, allow for the production of high‐quality crystal wafers with diameters up to 300 mm.

With a bandgap of 5.5 eV, compared to silicon's 1.1 eV, diamond is classified as a wide bandgap semiconductor. The large bandgap results in a comparably low intrinsic carrier concentration and correspondingly low thermal noise, which improves signal‐to‐noise ratio and stability. The caveat, though, is that it takes 13 eV to create an electron‐hole pair compared to the 3.6 eV required for silicon, which translates to a reduced sensitivity to ionizing radiation. This lower sensitivity should be considered carefully when choosing the base material within the context of a specific application.

The radiation hardness of diamond is largely due to the tetrahedral lattice structure and strong covalent bonding via sp^3^ hybridization. The bonds are strong (3.6 eV) compared to silicon (1.12 eV). In addition, the energy required to fully displace a carbon atom (42 eV) is significantly greater than the energy required to dislocate a silicon atom (13 eV). The majority of research is generally in agreement that diamond has superior radiation hardness when compared with silicon.^[^ ^89^ ^]^ It is worthwhile noting, that there are instances where silicon has been reported to have a superior radiation hardness compared to diamond.^[^ ^92^ ^]^ Therefore, the radiation environment, device architecture and sample quality should be well considered.

Diamond is often characterized as a tissue‐equivalent material, especially in comparison to silicon. For photons, this is largely due to the close match in atomic number of carbon (Z = 6) to soft tissue (Z_eff_ = 7.42), especially in comparison with silicon (Z = 14).The tissue equivalence of diamond with respect to charged particles, can be visualized by inspection and comparison of the stopping power ratios for diamond/water and silicon/water, provided in **Figure** **7**.^[^ ^93^ ^]^ Clearly, the diamond/water ratio is more stable than the silicon/water ratio over the given energy range. Whilst the ratio is not equal to one, the stability of the ratio over the energy range is suggestive that the response could be made to be equivalent given a suitable correction factor. Using this ratio as a basis, a dedicated Monte Carlo simulation study using the Geant4 toolkit was performed to determine if a geometric scaling factor could be used to mimic the response of charged particles in water for diamond. Two studies were conducted as part of this investigation. The first study was solely concerned with protons and alpha particles typical of both solar particle events and galactic cosmic rays.^[^ ^94^ ^]^ In the second study, the investigation dealt with heavy charged ions (2 ⩽ *Z* ⩽ 26) that form a minor if not insignificant component of galactic cosmic rays.^[^ ^95^ ^]^ In both studies, it was shown that to mimic the response within a cubic volume of diamond with a side length of 10 μm, a cubic volume of water with a side length of ≈ 31 μm is required. These studies demonstrated that a suitable geometric scaling factor can be applied to correct the response in diamond to water for charged particles, making diamond a suitable candidate material for solid‐state microdosimetry.

At CMRP, research on diamond‐based radiation detectors has primarily focused on radioprotection and medical applications, such as dosimetry and quality assurance (QA). In the field of radioprotection, diamond has been investigated as a base material for the development of solid‐state microdosimeters. Several proof‐of‐concept prototypes were developed in partnership with the University of Melbourne,^[^ ^94^, ^96^, ^97^ ^]^ highlighting the advantages and limitations of various detector architectures, ranging from simple planar metal‐insulator‐metal structures to more complex 3D configurations.^[^ ^95^ ^]^

The first prototype developed in this work is essentially a metal‐insulator‐p‐type diamond (m‐i‐p+) structure.^[^ ^96^ ^]^ As part of the fabrication process, a 2 MeV boron ion beam at a fluence of 1 × 10^15^ B cm^−2^ was directed onto the diamond surface through a 4 μm thick aluminium mesh to create a patterned electrode at a depth of 1.38 μm within the diamond, serving as a buried conductive electrode. The goal of this process was to create 3D vertical wall‐less SVs embedded in a diamond matrix. To establish a conductive pathway to this buried electrode structure, focused ion beam (FIB) milling was used to create a channel, which was then electroplated with gold/titanium to create the ‘back’ contact. A 0.5 μm aluminium pad was evaporated onto the surface to form the ‘top’ contact. To mitigate the effects of radiation damage upon the sample, annealing was performed after ion implantation and FIB processing. To evaluate the technology, the device underwent charge collection characterization using the Ion Beam Induced Charge (IBIC) technique with a 5.9 MeV beam of beryllium ions. The maximum charge collection efficiency (CCE) of this device was found to be approximately 30%. It is unclear whether this low CCE was due to radiation‐induced defect traps from the implantation process that were not removed during annealing, or other possible factors. Regardless, the low CCE indicates that the fabrication process requires further refinement.

The second structure developed was a simple metal‐intrinsic diamond‐metal (m‐i‐m) architecture.^[^ ^94^ ^]^ The primary purpose of this device was to explore the merits of physically isolating SVs through laser ablation. To investigate this, two sets of 2x2 arrays of aluminium contact pads (200 nm thick) were evaporated onto the top surface, with a common back contact of Ti/Au (20/150 nm thick). Laser ablation was used to create trenches 20 μm deep and 20 μm wide, surrounding the individual Al pads (600 × 600 $\;\mu m^{2}$) for one of the 2 × 2 arrays. The charge collection characteristics of the device were tested with 1.5 MeV protons and 5.5, 6.5, and 12 MeV alpha particles. The CCE of this device was found to be approximately 100%, indicative of the sample's quality. To test physical isolation versus electrical isolation, the device was operated in two different configurations, with and without the adjacent pads under bias. The key finding of this study was that physical isolation, combined with optimizing the electric field structure due to adjacent SVs, is the most efficient way of confining charge collection to the SV region.

The third structure, termed the ‘3D‐LES’ or 3D lateral electrode structure,^[^ ^97^ ^]^ investigated the viability of lateral structures as opposed to the previously described planar structures. Laser ablation was used to mill wells for the contact electrodes with dimensions of 80 × 60 × 30 $\;\mu m^{3}\;$. Electrode pairs were separated by distances of 10, 20, or 30 μm. Each well was then filled with a silver active brazing alloy to create electrode pairs. Electrode pairs were surrounded by isolation trenches, 5 μm wide and 60 μm deep. The CCE was assessed by an IBIC study with 5.5 MeV alpha particles, achieving a maximum CCE of 98%. Similar to the previous structure, the trenches assisted in charge confinement as long as the range of the ion species was less than the trench depth.

In the context of microdosimetry, the 3D structures were found to be the most effective. However, all devices, regardless of their structure, exhibited a critical flaw: undesirable charge collection from outside the SV. This issue arose because the device structures were fabricated on samples that were too thick for the intended application (500 μm), coupled with diamond's high charge carrier mobility and extensive charge collection distances. To enhance the confinement of charge collection, the primary recommendation was to develop structures on thin diamond films or to grow diamond to the required thickness directly on a suitable substrate. Each approach presents its own challenges: thin films (5–10 μm) are extremely fragile and difficult to handle, while growing diamond directly onto a substrate introduces the risk of contaminants entering the diamond or the chamber, potentially affecting subsequent growths. The authors believe that if the controlled growth of electronic‐grade diamond on suitable substrates can be achieved at a reasonable cost, then diamond could more effectively compete with silicon.

Following these early proof‐of‐concept devices, the development of diamond‐based microdosimeter technology has become more widespread.^[^ ^98^, ^99^, ^100^ ^]^ Perhaps one of the most interesting developments that has arisen is the microdosimeter developed by Zahradnik, et al, 2018.^[^ ^99^ ^]^ In this work, the authors report upon the fabrication and characterization of a single‐crystal chemical vapor deposition (scCVD) m‐i‐p+ diamond microdosimeter on a thin diamond membrane. A variety of processes, including cutting/polishing and Dry Reactive Ion Etching is used to best effect to create a thin diamond membrane (⩽ 10 μm thickness) within the sensitive region, surrounded by a thick (20 and 60 μm) diamond handle to maintain a semblance of mechanical stability. In addition, the process makes use of a thin, highly boron‐doped layer of diamond on the top surface which is then patterned to create an array of SV's with a built‐in bias due to the presence of the p+ diamond. Each SV is a rectangular prism with depth dependent on the thickness of the membrane and a minimum cross‐sectional area of 25 × 25 $\;\mu m^{2}$. Metalization of Aluminium onto the top and back side of the device allows for electrical connection and read‐out. This device is the closest analogue to the SOI microdosimeters that have been developed by CMRP,^[^ ^101^ ^]^ which has been constructed to date. If the next generation can reduce the size of SV's further, then it should satisfy many of the requirements of a diamond‐based microdosimeter.

An alternative research pathway at CMRP, has focused on investigating the merits and limitations of commercialized diamond‐based radiation detectors, such as the PTW microDiamond, for QA in both conventional and emerging radiotherapy techniques. The PTW microDiamond (PTW 60019, PTW, Freiburg, Germany) is perhaps the most well‐known diamond‐based radiation detector used in clinical RT. This detector utilises a cylindrical scCVD diamond with a diameter of 2.2 mm and a SV with a thickness of 1 μm. The architecture of the device shares similarities with the device described by Zahradnik et al. (2018),^[^ ^99^ ^]^ featuring a metal/intrinsic diamond/p‐type diamond (m‐i‐p+) structure. The inclusion of boron‐doped diamond in the structure creates a built‐in potential that promotes charge carrier drift without the need for an external bias, enabling passive mode operation.

A CMRP study examined the use of the PTW microDiamond in the edge‐on orientation for small field dosimetry.^[^ ^102^ ^]^ Previously, the edge‐on orientation had predominantly been used for QA in synchrotron‐based MRT.^[^ ^95^, ^103^ ^]^ Due to the IAEA TRS‐483 code of practice, this orientation had not been explored for conventional radiotherapy. However, the results of this pivotal study demonstrated the feasibility of the PTW microDiamond for QA in small field dosimetry. While diamond exhibits a near energy‐independent response with respect to water for x‐rays,^[^ ^104^ ^]^ the detector's housing and its high‐Z components can lead to density perturbation effects, commonly known as the stem effect, which can cause undesirable deviations in response. It is common practice to take such effects into account, however, when performing routine QA in clinical dosimetry, and so this issue is not novel. The next step is to determine output factors for the edge‐on orientation before clinical use is considered. An additional study was performed to make use of the fast signal collection due to its charge carrier mobility, charge carrier lifetime, and saturation velocity.^[^ ^95^ ^]^ The conventional mechanism for signal readout in a clinical setting is the use of an electrometer such as the PTW UniDOS. In this study, the purpose was to make use of the X‐TREAM data acquisition system^[^ ^105^ ^]^ and its MHz sampling rate to perform real time analysis of the pertinent features of an MRT treatment field, including but not limited to measurement of the FWHM and PVDR, which was successfully achieved.

Certainly, diamond has a role to play in the future of radiation detector for a variety of applications. While it seems unlikely that it will ever replace silicon entirely, except for niche applications, CMRP investigations are continuing.

### 3.5 Organic Semiconductors

The history of organic materials for radiation detection began as early as 1982 when the radiation induced change in electrical properties of polyacetylene was investigated using an electron beam, discovering that the electrical conductivity decreased slightly, while the activation energy increased slightly.^[^ ^106^ ^]^ Many teams, ranging from those in medical dosimetry through to high‐energy physics have since investigated the use of these materials due to their low manufacturing cost, their ability to be printed relatively easily as thin films using existing machinery^[^ ^107^, ^108^, ^109^ ^]^ (though optimizations for large scale mass manufacturing are required), and perhaps most importantly, their near water equivalence due to an effective Z very close to that of water. This water equivalence allows the use of a single calibration factor across different beam qualities and ensures the field is not perturbed by the measuring device.^[^ ^110^ ^]^

Prior research conducted for the purpose of creating organic solar cells and transistors, determined that the physics of charge generation and transport is unlike that of their inorganic counterparts and governs material selection and use.^[^ ^111^, ^112^, ^113^ ^]^ Organic materials possess an average dielectric constant of approximately 3, compared to that of about 12 for silicon. Hence, as shown in Equation (1), where ϵ_*r*_ is the dielectric constant of the material, organic excitons exhibit a much stronger Coulomb attraction potential whereby the electronic states are localised to individual molecules instead of the bulk solid.

(1) $$V=\frac{e^{2}}{4\pi \varepsilon_{r}\varepsilon_{0}r}$$

A consequence of this is that the exciton dissociation yield becomes a critical quantity for efficient charge extraction. Considered to be below the value required for considerable photon absorption,^[^ ^114^ ^]^ the dissociation length is in the order of 5 to 100 nm depending on the material,^[^ ^113^, ^115^ ^]^ though improvements have been presented in recent times.^[^ ^116^, ^117^ ^]^ So, while a desirable property of organic semiconductor detectors is that they can be manufactured into thin films, this thinness is actually a requirement for such a device and considerations must be made to ensure a measurable response.

Once excitons are created, the charges transport through the material by phonon‐assisted tunnelling from occupied localised states to nearby unoccupied localized states. However, as most organic materials are electrically neutral with no intrinsic potential, charges diffuse randomly through the device. This dictates the requirement of an external potential to bias the device and influence charges to diffuse towards the electrodes. To assist in overcoming these challenges, often a bulk heterojunction (BHJ), the mixing of n‐type and p‐type material at the molecular level, is preferred to increase the distance over which excitons combine by ensuring acceptor and donor states are always within the range of exciton dissociation.^[^ ^110^, ^118^ ^]^ These structures exploit the energy difference between the highest occupied molecular orbital and lowest unoccupied molecular orbital,^[^ ^119^ ^]^ analogous to the shifting of valence and conduction bands of an inorganic p‐n junction as the fermi levels reach equilibrium. These materials can then be structured in multiple thin‐film layers, sometimes including organic electret materials to create an intrinsic potential,^[^ ^120^ ^]^ allowing the production of organic BHJ diodes, and organic field effect transistors, general examples of which are displayed in **Figure**  **8**a.

However, the mechanisms described above are molecular level effects, whereas the quantity of interest, ionization, occurs on the atomic level. It is for this reason that many emerging devices are being used for indirect detection in the presence of a scintillator producing visible light, instead of direct detection. Previous work by CMRP showed that an organic photodiode, operated as a direct detector showed a nearly twofold over‐response during the measurement of a percentage depth dose distribution.^[^ ^121^ ^]^ The point of maximum dose was measured at almost twice the depth of the true location, when compared to validated reference dosimeters, as seen in Figure 8b. This was determined to be caused by a combination of the dose per pulse dependent response shown in Figure 8c, with and without the presence of a scintillator, and dose‐enhancement caused by other materials surrounding the organic photodiode. Once the device was coupled to a 1 mm scintillator, this effect disappeared and the device performed as expected, reproducing the percentage depth‐dose distribution measured with a calibrated ionization chamber, as shown in Figure 8d. The percentage depth‐dose distribution was consistent between measurements with a previously unirradiated device, as well as after being subjected to 40 kGy. Also discovered was the existence of a so‐called priming effect, whereby the signal produced during direct detection undergoes a delay in the order of seconds. The electrons generated by ionization within the device are not believed to generate a measurable current within this timeframe from the beginning of irradiation. Again, the use of a scintillator to operate the device as an indirect detector improved results; the rising edge of the signal improved significantly, representing the true dose being delivered to the detector. These results suggest that the future of organic semiconductors may exist as coupled devices utilizing an onboard scintillator within the packaging of the device, to ensure efficient use of the unique charge generation and transport properties described above.

The production of organic semiconductor devices as thin films allows them to be printed using existing manufacturing processes. Many materials can be dissolved into dye and manufactured into thin film structures, including pixelated arrays, using conventional printing processes. The nature of such thin, printed devices, often coupled with flexible substrates such as polyethylene (PET) allows for the production of flexible, wearable devices, utilizing existing large‐scale manufacturing such as low‐cost roll to roll printing technology.^[^ ^107^, ^122^, ^123^ ^]^ However, PET based materials have been shown to scintillate, exhibiting strong fluorescence in the visible portion of the spectrum,^[^ ^124^, ^125^ ^]^ the region which organic semiconductors are highly sensitive to. In light of this property, CMRP investigations into non‐fluorescent, conductive kapton substrates have been performed, resulting in flexible, tissue equivalent organic detectors with high stability.^[^ ^126^ ^]^ The ability to create thin, flexible, pixelated arrays that produce accurate results, presents the opportunity to monitor regions of the body during treatment with a single multi‐channel detector, while adhering to the contours of the patient.

Investigations probing the radiation hardness of organic semiconductors have not succeeded to produce a model that can be applied for all materials and situations.^[^ ^110^ ^]^ These studies have, however, produced promising results. One such study showed that a completely organic BHJ photodiode exhibited a stable response to radiation for the first 3 kGy, before which the response dropped to 30% after 35 kGy of absorbed dose. However, at this point the response again stabilized during measurements up to 40 kGy.^[^ ^121^, ^127^ ^]^ A similar phenomena was also observed under x‐ray irradiation of organic BHJ solar cells.^[^ ^128^ ^]^ A reduction in the photocurrent spectral response was found for doses up to 3 kGy, with a stabilization of the response after 10 kGy. Such behavior suggests that organic semiconductors may be tolerant to profoundly large radiation doses if a preirradiation step is performed.

While pixelated arrays may offer a solution for spatially focused dosimetry, often, vastly higher resolution is needed. In the case of MRT, previously described in Section 3.4, accurately measuring the dose within a 50 μm beamlet requires a device much smaller to avoid volume averaging effects. To exploit the extremely thin geometry of organic semiconductors, a device with a 500 nm thick sensitive volume was tested in edge‐on configuration at the Imaging and Medical Beamline of the Australian Synchrotron.^[^ ^127^ ^]^ Using three different energy configurations of the beam, where each possesses a different dose‐rate, the device accurately reproduced the spatial characteristics of the beam for the highest dose‐rate. For the two lowest dose‐rates, the FWHM was not in agreement with known beam parameters. This was believed to be due to previously discussed effects of dose‐rate upon organic semiconductors operating in a direct detection configuration. In addition to the reported parameters, the shape of the microbeams deviated from the expectation of sharp, single peaks,^[^ ^105^, ^129^ ^]^ with a shoulder appearing as a smaller, secondary peak to each central maxima. A hypothesis that this was due to the presence of PET in packaging material, causing fluorescence as previously discussed, was confirmed by reversing the orientation in which the beam was scanned, as well as a Monte Carlo simulation utilizing the Geant4 toolkit. As these mechanisms are understood, further work is continuing for the use of these detectors for ultra‐high spatial resolution, and wearable, tissue equivalent dosimetry.

### 3.6 Scintillator Fiber‐Optic Detectors

In parallel with utilizing electronic properties of semiconductor materials to create devices for radiation dosimetry, photonics‐based dosimeters have also been widely developed and applied for the same purpose. Fiber‐optic dosimeters (FODs) represent a significant advancement in photonics‐based technology. Taking advantage of novel scintillation materials, the availability of fiber optic components and optoelectronic devices, allows the design of novel devices that provide precise real‐time radiation measurements in various challenging environments and conditions.^[^ ^130^ ^]^

A FOD consists of a small volume scintillator and an optical fiber that transmits the emitted scintillation light to an optical detector, converting the optical signal to an electronic signal, which can be subsequently captured with a data acquisition system. The optical fibers used to construct FODs most often consist of poly methyl methacrylate or silica; therefore, they are often considered to be water or tissue equivalent for dosimetry in radiation therapy. The scintillating material that generates the light to be transported by the FOD can take the form of either a small plastic scintillator, single crystal inorganic material, scintillating powder encased within a polymer, or even a small piece of scintillating optical fiber, manufactured using either an organic or inorganic scintillating material.

Such devices present many advantages for dosimetry in medical radiotherapy, including the aforementioned water/tissue equivalence (especially when coupled to plastic scintillators), compact sensitive volumes, high sensitivity, real‐time response, angular independence, and an absence of electrical interference, as all electronics are external to the radiation field. These features make FODs an attractive option for verifying the dose delivered within complex external beam treatment techniques, such as intensity‐modulated radiation therapy, stereotactic body radiotherapy, and volumetric‐modulated arc therapy. FODs are also emerging as a potential solution to challenges found in new treatment modalities such as proton and heavy‐ion therapies, as well as FLASH where extremely high dose‐rates are utilised.^[^ ^131^, ^132^, ^133^ ^]^ However, the fast‐track development of tissue equivalent FODs in radiotherapy has been limited somewhat by their radiation related challenges. These include a strong LET dependence in mixed radiation fields due to scintillation light quenching, Cherenkov light production in the fibers and its dependence on the incidence angle of the irradiation,^[^ ^134^, ^135^ ^]^ total irradiation dose impacting the scintillation output as well as the radiation hardness of the optical fibers. The small sensitive volumes of FODs render them extremely suitable for small‐field dosimetry where the size of the device must be much smaller than the lateral extent of the radiation field, and they may also be employed during in vivo dosimetry. These small sensitive volumes are usually on the order of 1  mm^3^ , hence, they can be treated as point detectors.

CMRP developed FODs have been successfully used in the measurements of synchrotron microbeams with highly impressive resolution, as low as 10 μm in one direction.^[^ ^136^, ^137^ ^]^ Such devices have also proven to be particularly useful for Magnetic Resonance‐guided Linear Accelerator (MRI‐LINAC) dosimetry since they are theoretically immune to density induced fluence perturbations found in MRI‐LINACs. Simultaneously, the non‐ferromagnetic compositions of FODs allow for real‐time use without inducing perturbations of the magnetic field produced by the MRI scanner, a critical benefit for patient specific quality assurance dosimetry.^[^ ^138^, ^139^, ^140^ ^]^

Spurred from the research and development of FODs at CMRP, as well as their potential applications, a wide range of research interests in multiple areas have evolved, including but not limited to, material science of scintillators, design and fabrication of FOD probes, novel techniques for stem effect removal, and signal processing methodologies and algorithms.

## 4 Conclusion

While CMRP is an established, global leader in the development of crystalline silicon‐based radiation detectors, this concise review has described the caveats of a material that has served the field of radiation detection for many decades. Clearly, investigation into other materials is required, a direction that CMRP jointly pursues alongside our existing scope of research. Many of these materials offer solutions to challenges currently faced, solutions that may not be available otherwise. It should be clear, however, that a ‘one size fits all’ solution is unlikely to ever be achieved due to the extremely broad properties of the range of ionizing radiation fields, as well as the manipulation and delivery of these fields as new radiation oncology modalities emerge. Where one material may offer tissue equivalence superior to that of crystalline silicon, it may also suffer from a dose‐rate dependence. Another material may solve the challenges of ultra‐high dose‐rate measurements, though it exhibits less than desirable radiation hardness. As exciting new detector material developments, we have discussed several emerging candidates. a‐Si:H possesses high spatial resolution, high radiation hardness, passive operation, and angular dependence over a full rotation of a medical linac. However, charge extraction poses a challenge, as to be used for radiation dosimetry the sensitive volume must be manufactured thicker than for solar cells, the leading use of a‐Si:H. Similarly, SiO_2_, is used to produce MOS*kin* dosimeters that are dose‐rate independent, suitable for skin dosimetry, and have a highly controllable sensitivity rendering them suitable for all radiation types. The integrating MOS*kin* dosimeters do have a radiation lifetime that is limited relative to the total radiation dose often received in clinical treatments. However, these MOS*kin* detectors can be effectively reset with emerging annealing technology. When reading a detector in real‐time, low leakage currents and low noise, while remaining radiation hard are clearly desirable traits, of which SiC provides. However, the low electron and hole mobility may be the cause of volume‐averaging like effects. Diamond‐based detectors solve this challenge, but are significantly more expensive to produce, requiring extremely high purity samples to manufacture the sensitivity volumes. At first glance, organic semiconductors appear to solve all the previously mentioned challenges. However, the charge generation and transport intrinsic to these tissue‐equivalent materials are molecular‐level effects, not the atomic level at which ionization occurs. This can be solved with the addition of a plastic scintillator for indirect radiation detection, though this creates the possibility of measurement errors due to undesirable light production caused by packaging materials. Scintillating fiber‐optic devices offer a unique perspective relative to the previous materials, as they use light for signal transport, with all electronic circuitry outside of the radiation field. However, Cherenkov light production, reduction of scintillator efficiency and radiation hardness of the optical fibers present challenges for the widespread adoption of devices produced from these materials.

Like silicon, devices manufactured from the new materials discussed in this review are generally used as relative dosimeters, requiring calibration against accepted and highly regulated primary standards. With improvements in charge collection efficiency, in some cases up to 100% as seen within this review, significant progress can be made toward creating novel devices suitable for absolute dosimetry. CMRP is at the forefront of microdosimetry, for instance, currently producing silicon‐based devices with incredible accuracy, allowing spectroscopic measurements to allow determination of absolute dose at the cellular level. Clearly, new materials offer exciting solutions to existing hurdles and provide a secondary avenue to pursue alongside silicon for such devices.

For these reasons, CMRP continues to lead investigations aimed at unravelling the properties of these alternative materials. Concurrently, CMRP advances the frontier of end‐user device development, targeting solutions suitable for existing clinical and research environments, as well as hypothesized facilities that are yet to be developed, presenting a new set of challenges, and of course, opportunities.

## Conflict of Interest

Michael Lerch, Marco Petasecca, Dean Cutajar, and Anatoly Rosenfeld declare consultancy with Electrogenics Ltd, who are commercializing the MOSkin detector.
